# A novel self-seeding method for particle image velocimetry measurements of subsonic and supersonic flows

**DOI:** 10.1038/s41598-020-67680-5

**Published:** 2020-07-02

**Authors:** Omid Nematollahi, Hadi Samsam-Khayani, Mahdi Nili-Ahmadabadi, Sang Youl Yoon, Kyung Chun Kim

**Affiliations:** 1 School of Mechanical Engineering, Pusan National University, Busan, 46241 Republic of Korea; 2 Department of Mechanical Engineering, Isfahan University of Technology, Isfahan, 84156-83111 Iran; 3 Rolls-Royce and Pusan National University Technology Centre, Pusan National University, Busan, 46241 Republic of Korea

**Keywords:** Energy infrastructure, Mechanical engineering

## Abstract

A self-seeding particle method is proposed for particle image velocimetry measurements in closed cycles such as Organic Rankine Cycles. Condensed droplets of vapor are used as tracers in a closed cycle for both subsonic and supersonic regimes. A free jet of R245fa in the vapor phase is examined in a case study with two different nozzle pressure ratios of 5.1 and 2.1 to evaluate the tracer particles in both supersonic and subsonic conditions. A simple turbulent jet in subsonic conditions and an under-expanded jet are observed in high supersonic conditions. The flow structures of the under-expanded jet are captured using the proposed method, and vivid images of the Mach disk and shock cells are obtained. A series of Schlieren photography experiments are performed to validate the proposed method. The results show that the method can be a good candidate for tracer particles in the closed cycles where condensation of the working fluid is possible.

## Introduction

Organic Rankine Cycles (ORC) are one of the most notable methods for waste heat recovery for low to medium heat sources from several kW to MW. The heat source in this area include the renewable energy sources^1^, biomass^2^, geothermal^3^, and waste heat^4^. Expander plays the main role in ORC which can affect the overall efficiency and performance. The expander includes the turbomachines such as axial, radial and volumetric ones, where the working fluid is an organic vapor. Design of such turbomachines are based on the CFD modeling that properties of material modeled by different thermodynamic models^5,6^.

To validate the accuracy of such methods experimental data are needed. Up to date, detailed quantitative experimental results are not published in the literature due to challenges related to this field. However, a couple of results related to Schlieren measurements are published^7^.

There have been a couple of attempts for measuring the flow characteristics of this flow but they have remained on the design step or point measurement such as TU Delft (flexible asymmetric shock tube)^8,9^, Politecnico de Milano (test rig for organic vapors)^10^ and by White and Sayma^11^ where a closed-loop of organic vapors was designed. One of the important methods for quantitative measurements of flow structures of the desired flow is Particle Image Velocimetry (PIV).

Currently, solid and liquid particles are used as seeding particles in PIV measurements for both liquid and gaseous flows^12,13^. The appropriateness of tracer particles is dependent on the flow properties (the density, temperature, and flow regime). Generally, seeding for liquid flows such as water is much easier than for gaseous flows^14^. For liquid flows, particles with matched density are commonly used.

For a gaseous flow, both liquid and solid particles can be used as tracers. For atomization, different oils or di-ethyl-hexyl-sebacate (DEHS) can be used with a simple Laskin nozzle, which can produce particles with diameters of around 1 μm. For high-temperature applications with high-speed flows, solid particles like TiO_2_ and SiO_2_ are common and are commercially available.

The applications for compressible flow measurements include supersonic jets^15–17^, wind tunnel measurements^18,19^, and turbomachinery flows^20^. However, all the related compressible flows have been examined in an open-cycle wind tunnel or closed-cycle airflow with solid particles so far. Seeding liquid particles in a wind tunnel requires frequent cleaning of the facility and visualization windows. Furthermore, in the case of solid particles, the accumulation of particles inside the tunnel should be considered^11^. Another difficulty of seeding air flow is that the flow should not be disturbed by the particles, and the uniformity of particles must be kept constant.

There are other challenges for closed cycles such as the organic vapor cycle. For example, air should be evacuated from the test rig. Therefore, a particle-seeding method associated with air cannot be used. Thus, the Laskin nozzle method for liquid tracers is not feasible. In addition, if the closed cycle can reach high temperatures, then a method with oil is not possible due to the break-up of the oil particles. Adding such droplets can also change the main fluid properties, which is not desirable, and the oil droplets create a film inside the cycle and test-section windows.

Solid tracer particles such as TiO_2_, SiO_2,_ and Al_2_O_3_ have several challenges^21^. In addition to accumulating inside the test setup, purging and filling the refrigerant requires extra time, labor, and cost. Another issue comes from rotary equipment inside the cycle, such as expanders, turbines, and compressors, which can be damaged by the particles. In addition, cleaning and purging the cycle are required after the operation. Changing the properties is not significant in this case, but the accumulation of particles inside the reservoir tank can result in blockage due to agglomeration.

In conclusion, following all the limitations of such dense flows, providing a set of quantitative experimental results for validation of numerical simulations are necessary. To the best of the authors’ knowledge, tracer particles in such a closed cycle with an application for ORC have not been studied previously. In the current study, a novel method is proposed and evaluated for PIV measurement inside a closed cycle of organic vapor in a closed wind tunnel for both subsonic and supersonic regimes. This method can be used to prepare the experimental results of simple flow to validate the dense gas numerical simulations.

## Experimental setups

**Figure 1:**
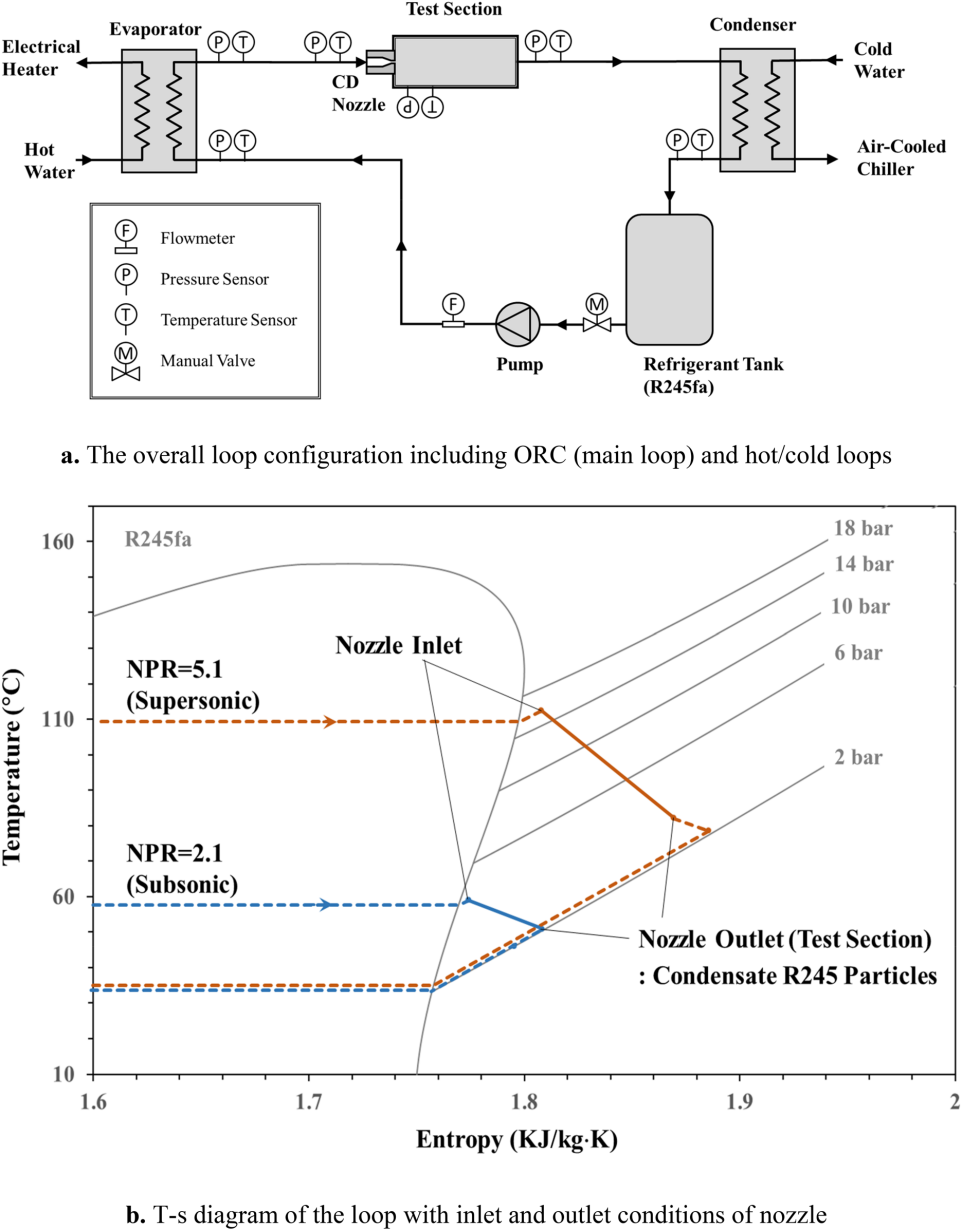
General test setup and T-s diagram of test points^23^.

To study the particle-seeding method, a closed cycle was designed with two hot and cold cycles in parallel, as shown in Fig. 1. As a case study of a closed-cycle wind tunnel, an organic Rankine cycle (ORC) with an organic fluid as the working fluid was selected for evaluation of the proposed method. The ORC performance in real conditions always has a superheating degree of 5 to 8 K based on the design criteria^22^. Therefore, the possibility of condensate particles occurring inside the gas flow should be evaluated. The proposed method uses condensate particles inside the gas as tracer particles.

To study the feasibility of the method, a jet flow of R245fa was considered in the closed cycle^23^. This method could be applied to other vapors where condensation is possible. A convergent-divergent (CD) nozzle was designed and implemented in the main cycle, as shown in Fig. 1a. Two different nozzle pressure ratios (NPRs) were applied to the CD nozzle to study the performance of the condensate flow as tracer particles in the subsonic and supersonic regimes. A T-s diagram of the test cases is presented in Fig. 1b.

**Figure 2:**
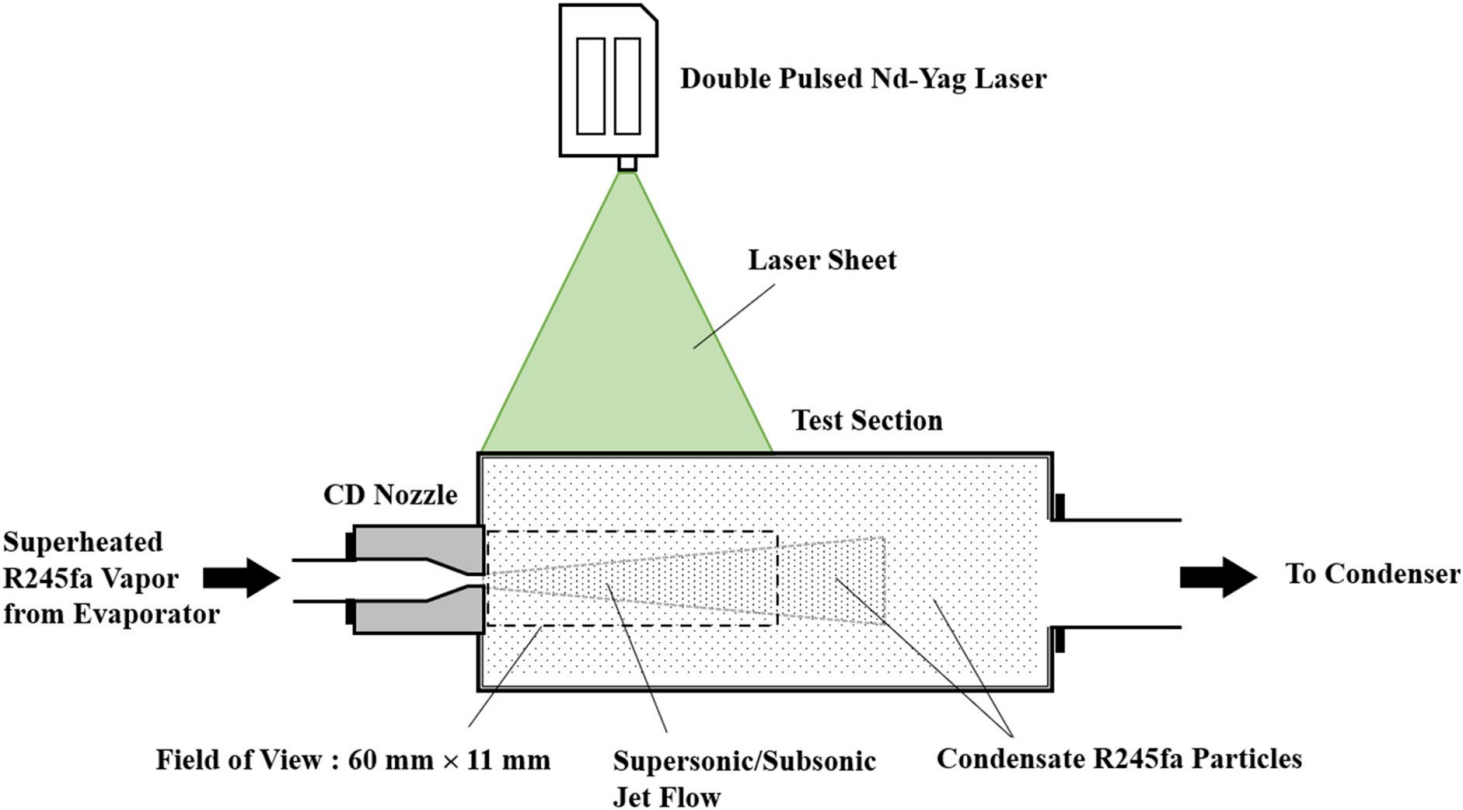
PIV configuration with details of experimental flow field.

The PIV configuration is shown in Fig. 2. A 2D PIV system was used to observe the condensate particles to trace the main flow. The PIV system consists of a PIVCAM 10–15 CCD camera, a double-pulsed Nd–Yag laser with a maximum power of 200 mJ/pulse, a TSI 610032 synchronizer, and a computer. The time difference between two laser pulses was 1 μs, and the frequency of the laser pulses was set to 3 Hz. The particle image size for the PIV measurement was 1,280 by 280 pixels. The size of the interrogation window for the velocity calculation was 32 × 32 pixels with a 50% overlapping. In-house software was used for PIV post-processing^23^.

**Figure 3:**
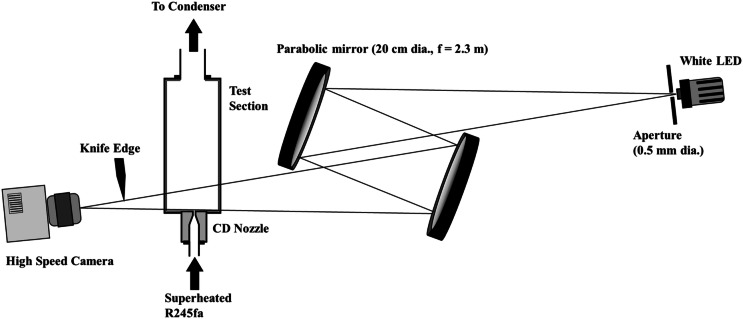
Schematic of modified Z-type Schlieren^24^.

To validate and visualize the flow structures of the free jet in detail a modified Z-type schlieren^24^ has been utilized. The modified version of Z-type Schlieren can be used where the space in the laboratory is limited due to normal Z-type Schlieren the distance between two PM should be 2 times the focal length of PM while the test section is located in the middle of PMs. Furthermore, as in this modified version, the ray path from LS to the camera is shorter than the conventional type, then the image quality is higher. This method was presented by Wu and New^24^ as shown in Fig. 3. The setup is included of two PM with 20 cm diameter while f = 2.3 m. A 2.5-W white LED (Thorlabs MCWHLP1) is used as the light source while a 0.5 mm aperture is used. A Phantom high-speed camera is used to capture the images.

## Proposed method and results

Measuring the response time of tracer particles is a challenging task for a number of reasons. The particle properties cannot be quantified directly, but for solid particles, an average can be obtained when they settle on support. Nevertheless, due to agglomeration in the flow, the uncertainty of the particle size should be considered^25^. Furthermore, the liquid particles can change in size during the experiment due to possible condensation and evaporation. Therefore, the tracer response time should be determined by evaluating the tracer particles in a test case^26^.

**Figure 4:**
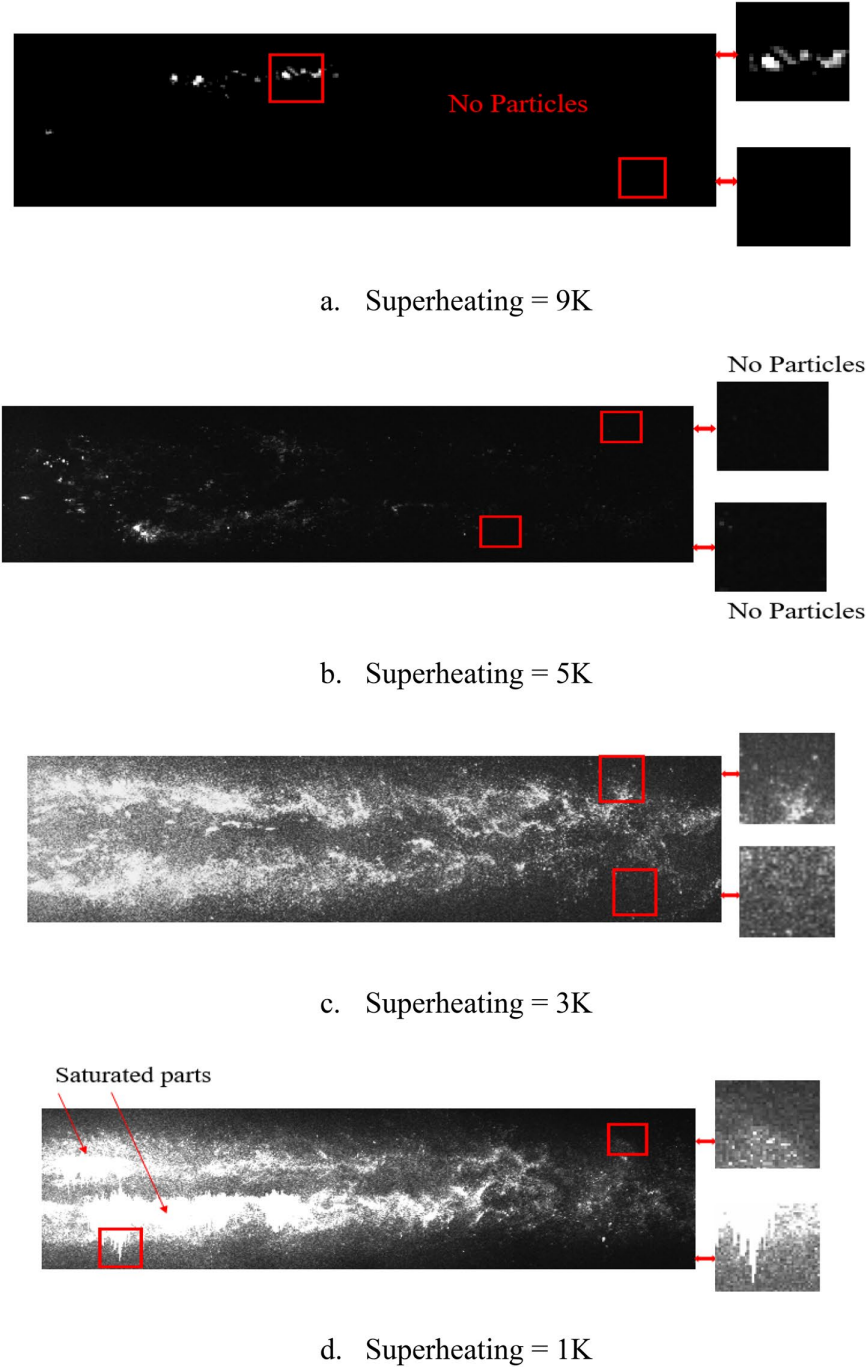
The effect of superheating degree on condensate particle density.

In the recommended superheating degree range of 5–8 K (due to ORC performance based on the recommendation of Nematollahi et al.^22^), condensate particles may be present in the main flow^23^. Figure 4 shows the particle distribution at different superheating degrees inside the test section.

**Figure 5:**
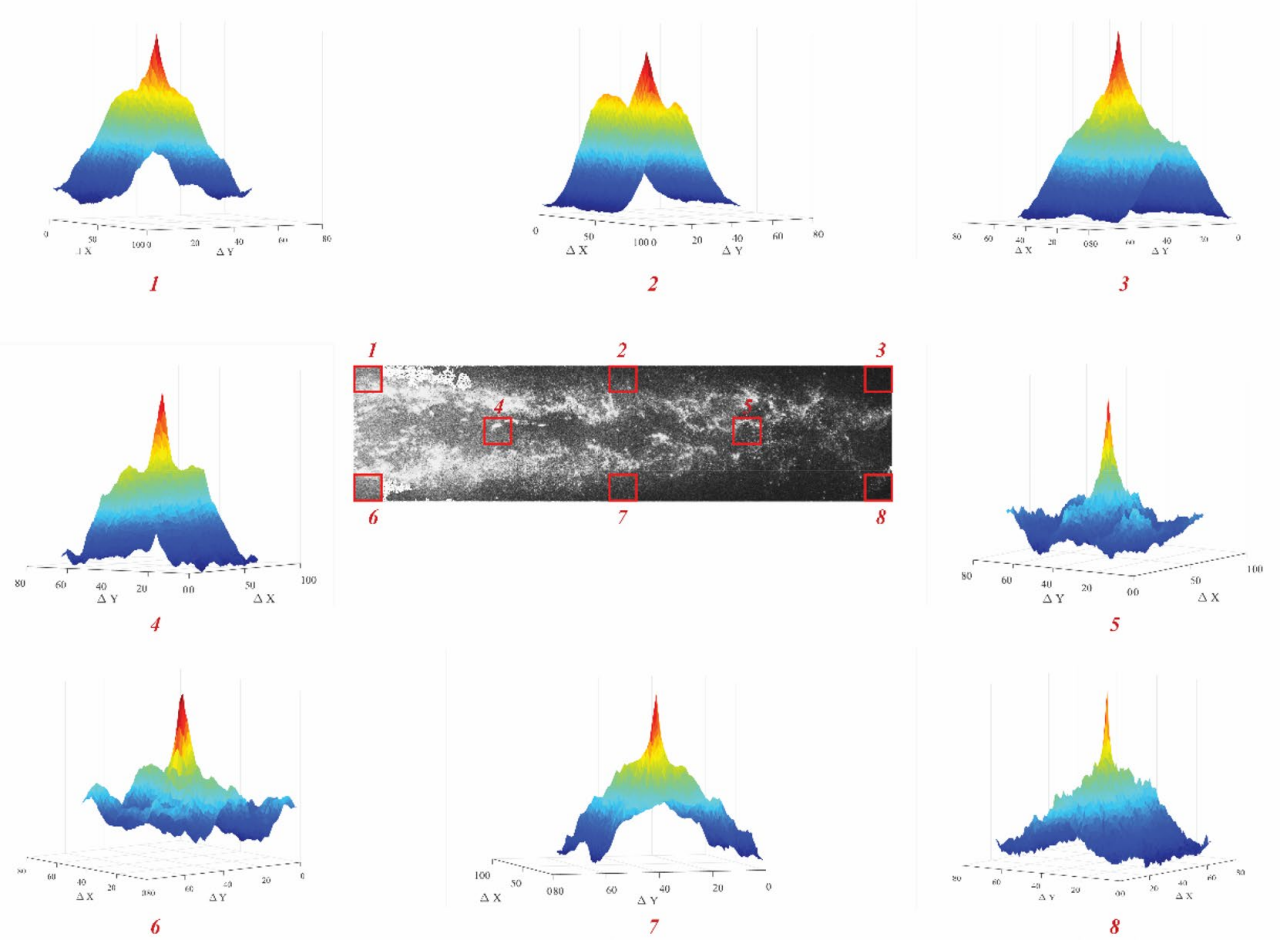
Cross-correlation maps for different location of Fig. 3c.

At a superheating degree higher than 5 K, the number of condensate particles decreased. Therefore, the amount of particles is suitable for PIV analysis. For the superheating degree of 3 K, the particles uniformly distributed inside the test section, and the density of particles was enough to capture images. However, for a superheating degree of 1 K or less, the droplets became larger and agglomerated more, so they cannot be used as tracer particles. In this range, the image will be saturated with light. Therefore, the particles obtained with a superheating degree of 3 K were used for the rest of the experiments. It should be mentioned that these particles are not visible with the naked eye when they are present in the main flow. In addition, Fig. 5 presents the cross-correlation maps for the instantaneous double-frame for a single interrogation region following Fig. 4c. The maps clearly show the pick of cross-correlation and proved the ability of this method for capturing the velocity of flow.

**Figure 6:**
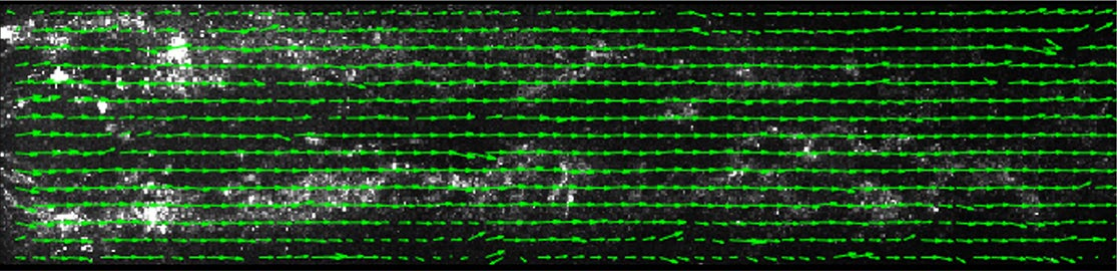
Raw image with superimposed vector field for 3 K of superheating (Fig. 3c).

**Figure 7:**
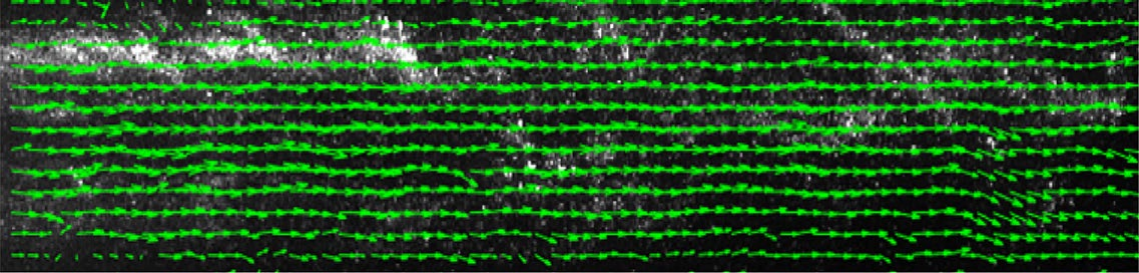
Raw image with superimposed vector field at 3 K of superheating in subsonic conditions (NPR 2.1).

Figure 6 presents the condensate particles with a superimposed instantaneous vector field at a superheating degree of 3 K. In addition, Fig. 7 shows the raw image superimposed with the corresponding instantaneous vectors for an NPR of 2.1 with a superheating degree of 3 K. The results show that condensate particles can capture the flow features well and instantaneously.

**Figure 8:**
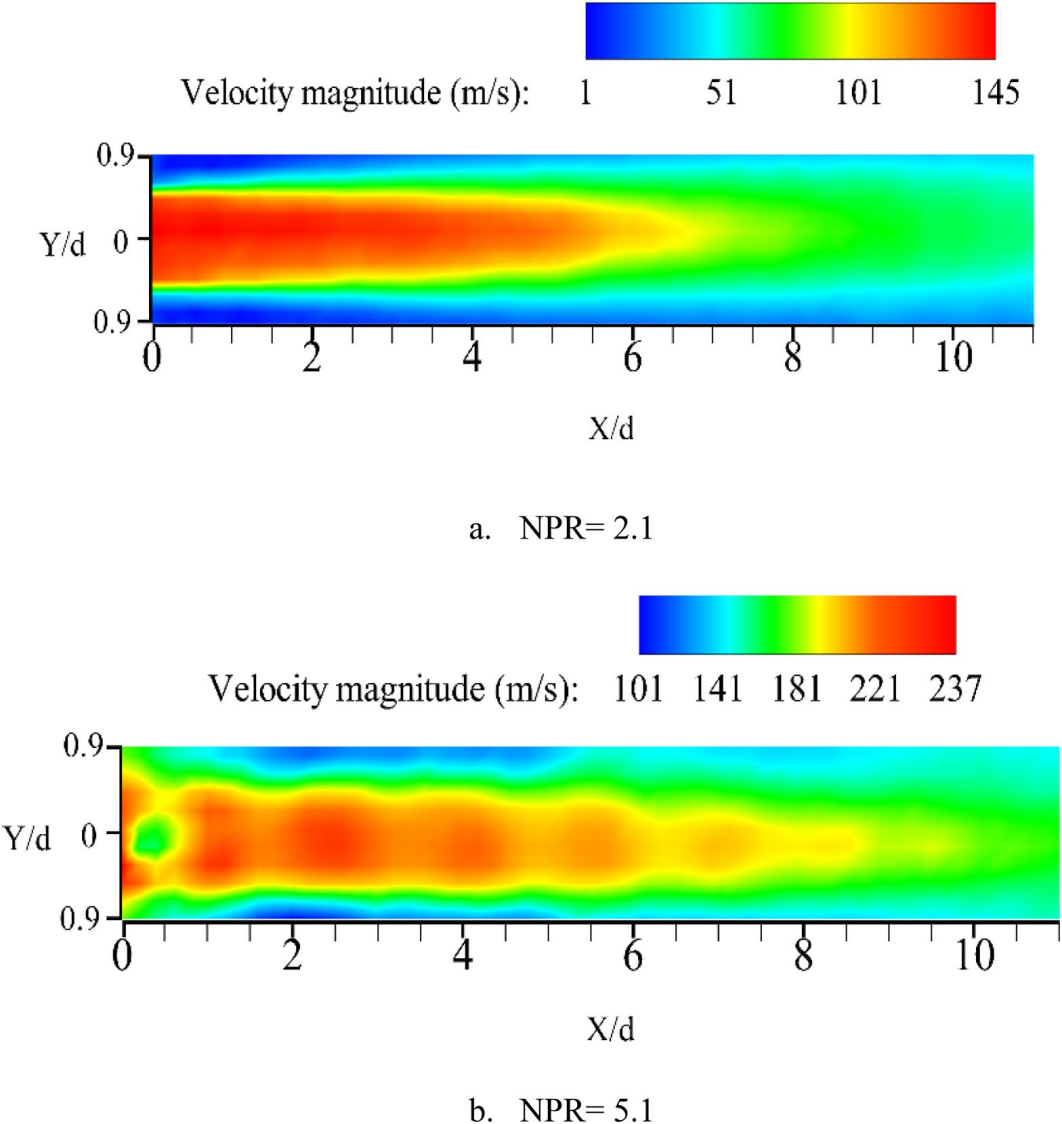
Averaged velocity magnitude contours illustrating subsonic and supersonic free jets.

Figure 8 shows the average velocity magnitude contours at NPRs of 2.1 and 5.1 in the outlet of the nozzle. The results show that the jet at NPR = 2.1 is a subsonic jet without any shocks. The shear layer was created between high-velocity regions in the core region of the jet. At NPR = 5.1, a highly under-expanded jet is formed. The average sound speed for these conditions is 180.5 m/s, which corresponds to a maximum Mach number of 1.3. The results clearly show the Mach disk, shock cells, and jet boundary, which includes the expansion and compression fan. The tracer particles captured the flow characteristics of subsonic and supersonic flows of the R245fa inside a closed cycle^23^. Therefore, the condensate particles are a good candidate for closed-cycle PIV measurements by controlling the superheating level.

**Figure 9:**
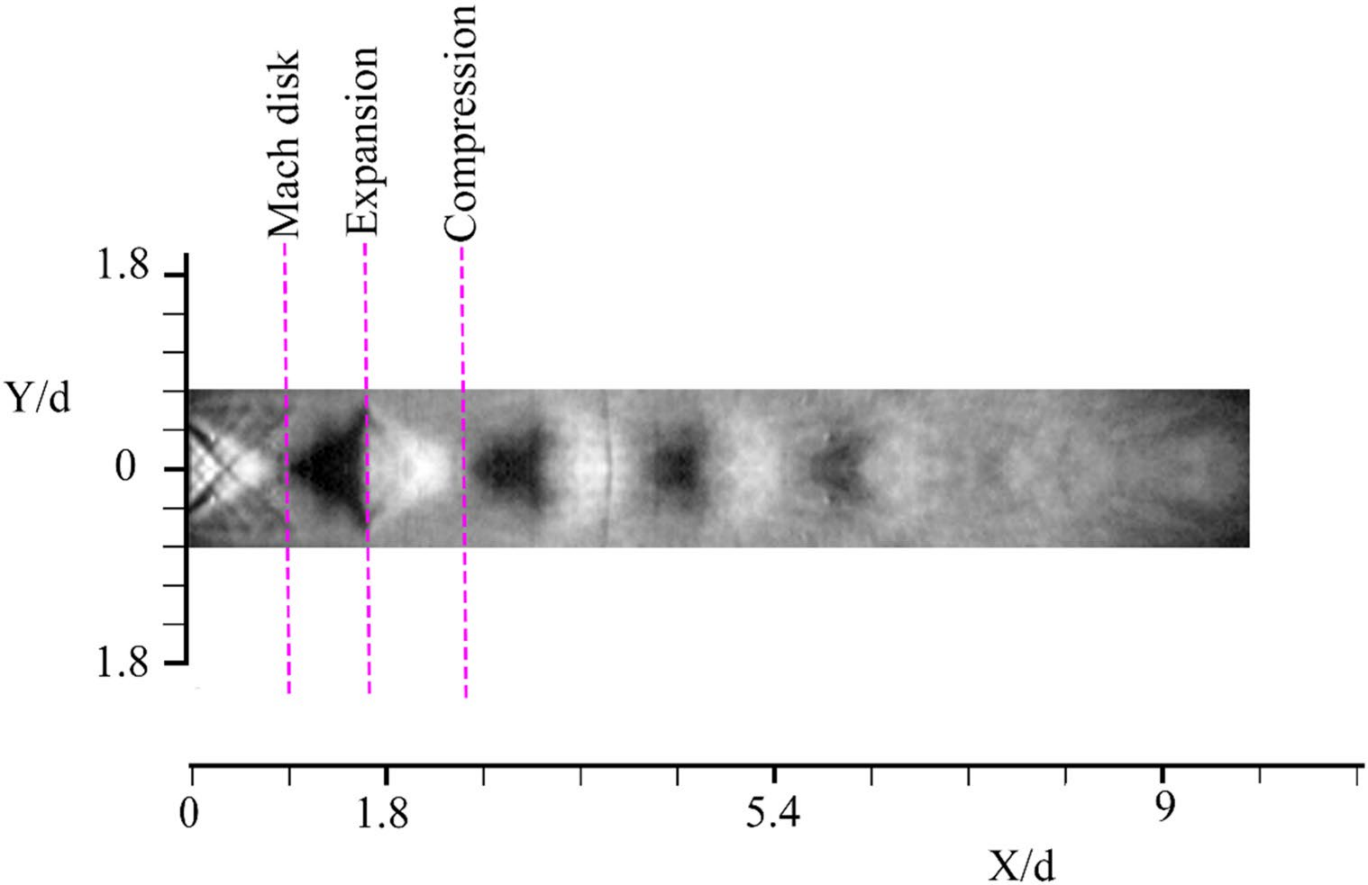
Schlieren photography using vertical KE.

To validate the PIV measurement method, a series of Schlieren visualization was made in the same condition for the supersonic case. Schlieren shadowgraph using a vertical Knife Edge (KE) is shown in Fig. 9 for supersonic condition. Using a Vertical KE (VKE) gives the density change clearer in the vertical direction. Furthermore, in VKE positioning the compression fans are clearer.

The comparison of Schlieren photography with PIV measurement shows the good agreement between both methods which are both qualitative and quantitative. As can be seen, the proposed method could capture even the abrupt change when shock diamonds occur. The PIV flow structures are the same as Schlieren photography which can prove the effectiveness of the proposed method with its uncertainties. Also, as mentioned earlier in the startup of this section, sometimes, the response time measurement of tracer particles is challenging. Therefore, the tracer response time should be determined by evaluating the tracer particles in a test case. The validation of the proposed method can be vivid evidence that the tracer response time is enough to capture flow structures even when abrupt changes in the velocity happen.

## Conclusion

In this paper, a self-seeding particle method was proposed for use in closed cycles in both subsonic and supersonic flow regimes. This method uses condensate particles of the main working fluid that are not visible by the naked eye. A case study of a free jet flow was done to evaluate the proposed method with ORC and a working fluid of R245fa in a closed cycle. The superheating degree of the main flow should be controlled to be lower than 5 K and higher than 1 K to obtain enough separated particles for use in PIV measurements. A superheating degree of 3 K was considered in this study.

The results at two NPRs of 2.1 and 5.1 showed that the condensate particles are good candidates for use as tracer particles in PIV measurements. The results in both subsonic and supersonic regimes revealed the relevant flow structures. Therefore, the current method is applicable for PIV measurements in a closed cycle.

Furthermore, even though an organic vapor was utilized for the evaluation, the method could be extended for all fluids that can undergo condensation. However, it should be mentioned that the proposed method faces a couple of limitation including superheating degree controlling and its limited temperatures span for any given pressure. Nevertheless, the proposed method is the more recent technique to measure the velocity of such flows.
